# Histopathologic and Molecular Insights Following the Management of Ameloblastomas via Targeted Therapies – Pathological and Clinical Perspectives

**DOI:** 10.1007/s12105-024-01734-2

**Published:** 2024-12-02

**Authors:** Ariel Hirschhorn, Shirly Grynberg, Gadi Abebe Campino, Alex Dobriyan, Vinod Patel, Gahl Greenberg, Rinat Yacobi, Iris Barshack, Ran Yahalom, Amos Toren, Marilena Vered

**Affiliations:** 1 Department of Cranio-Maxillofacial Surgery, Chaim Sheba Medical Center, Tel Hashomer, Israel; 2 Ella Lemelbaum Institute for Immuno-Oncology, and Melanoma, Chaim Sheba Medical Center, Tel Hashomer, Israel; 3 Division of Pediatric Hemato-Oncology, Edmond and Lily Safra Children’s Hospital, Sheba Medical Center, Tel Hashomer, Israel; 4 Department of Oral Surgery, Guys & St Thomas Hospital, London Bridge, London, UK; 5 Department of Diagnostic Imaging, Sheba Medical Center, Tel Hashomer, Israel; 6 Institute of Pathology, Sheba Medical Center, Tel Hashomer, 5265601 Israel; 7 Department of Oral Pathology, Oral Medicine and Maxillofacial Imaging, School of Dentistry, Tel Aviv University, Tel Aviv, Israel

**Keywords:** Ameloblastoma, BRAF mutation, Targeted therapy, Response, Epithelium, Stroma

## Abstract

**Purpose:**

Current standard of care for ameloblastoma (conventional/unicystic - mural type) usually mandates extensive bone resection that frequently necessitates immediate reconstruction with serious sequelae, especially among young patients. *BRAF*-mutated ameloblastomas can be targeted by BRAF inhibitors to markedly reduce their size, enabling conservative removal of residual tumor. We aimed to characterize the effect of post-treatment histomorphologic changes.

**Methods:**

Study included 14 patients, 11 mandibular and three maxillary tumors. Cases with very minimal residual tumor were defined as near-complete response, while those with mostly vital residual tumor as partial response. The epithelium component was scored for architectural and cellular changes, stroma - for fibrosis, inflammation and new bone formation, on a 3-tired score system: 0–no, 1–focal and 3–frequent changes. The mean scores of each parameter, total epithelium and total stroma were calculated and related to duration of treatment. Differences in the mean scores were investigated for mandibular tumors with near-complete response (*n* = 3) and partial response (*n* = 8).

**Results:**

There were no significant differences in mean epithelium or stroma scores between tumors with near-complete and those with partial response (2.22 *±* 0.68 *versus* 2.08 *±* 0.43, *p* = 0.55; 1.41 *±* 1.04 *versus* 1.43 *±* 0.44, *p* = 0.27), suggesting that ameloblastomas have potential to undergo complete response to targeted treatment. This is probably dependent upon tumor/patient/treatment-related factors. Response to treatment appears to be predictable with neoplastic epithelium being first, while the stromal response increases during treatment, the entire process expanding over weeks-to-months.

**Conclusion:**

Albeit preliminary, these are the first comprehensive histomorphologic findings on BRAF-treated ameloblastomas. Analyzing the suggested parameters in tumors with partial response, should highlight which tumor component has responded/failed to respond. This could serve as a basis for decision-taking toward subsequent steps in adjuvant treatment (e.g., follow-up, conservative surgery, modifications/changes in treatment regimen, combinations of approaches), with a prime aim of jaw preservation and minimal risk of sequelae.

**Supplementary Information:**

The online version contains supplementary material available at 10.1007/s12105-024-01734-2.

## Introduction

Ameloblastoma of the jaws is the most common epithelial odontogenic tumor. It is sub-classified into intra-bony conventional and unicystic varieties. The unicystic ameloblastoma comprises of luminal, intraluminal and mural subtypes [1, 2]. Ameloblastomas have no cytological atypia, are defined as benign even though they may display locally aggressive biological behavior [1, 2]. The current standard of care mandates bone resection with a 1–2 cm margin of safety beyond the radiological borders for both the conventional and unicystic mural subtype, due to similar risks of recurrence [2]. The resulting bony defect frequently calls for immediate reconstruction, which is usually based upon a vascularized free flap harvested from an additional surgical site. These extensive surgical procedures might permanently affect jaw and tooth development, function, and esthetics, and inevitably have a negative psychological impact, especially among young patients [3]. Furthermore, such extensive surgery bears morbidity that might require additional surgical procedures. These sequelae mandate the search for enhanced treatment approaches.

Identification of the *BRAF*^*V600E*^ driver mutation in mandibular ameloblastomas as well as other mutations usually associated with maxillary ameloblastomas almost a decade ago has led to the search for targeted therapy for ameloblastomas of the jaws [4–6].

Reported attempts to treat ameloblastomas with BRAF inhibitors (BRAFi) [with/without mitogen-activated protein kinase (MEK) inhibitors (MEKi)] as a last line treatment, have been successful for seven patients with recurrent inoperable conventional ameloblastomas, even some with lung metastatic deposits [7–14]. BRAF-targeted therapy was used effectively for the first time in a neoadjuvant protocol in a small series of young patients with unicystic, mural type ameloblastoma [15]. Tumors responded with a significant reduction in size to a point that enabled conservative removal of the residual tumor, enabling jaw preservation and ad integrum mandibular regeneration. These young patients were part of a larger series of 11 patients (eight unicystic mural type, three conventional ameloblastomas), successfully treated with neoadjuvant BRAFi (with/without MEKi) [16]. An additional case of ameloblastoma with a short-term neoadjuvant BRAFi protocol has also been reported [17].

**Fig. 1:**
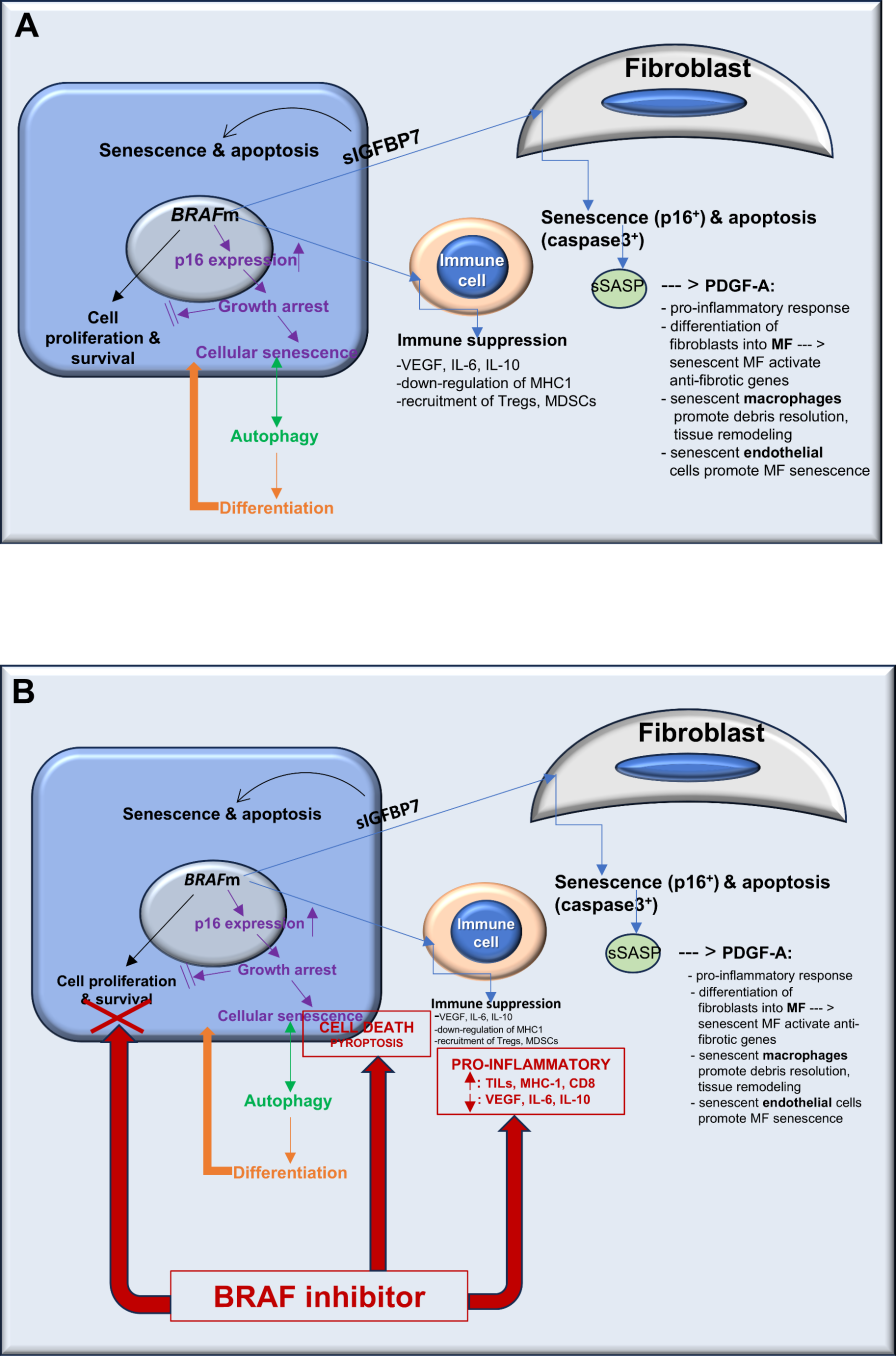
(**A**) *BRAF* mutation – main effector pathways in mutated cell and in stromal immune cells and fibroblasts; (**B**) BRAF inhibitor – main effector pathways in mutated cell and in stromal immune cells and fibroblasts. Abbreviations: BRAFm – BRAF mutation; CD – clone of differentiation; IL – interleukin; MCH – major histocompatibility complex; MDSC – myeloid–derived suppressor cell; MF – myofibroblast; PDGF – platelet–derived growth factor; sSASP – secreted senescence–associated secretory phenotype; sIGFBP7 – secreted insulin–like growth factor binding protein 7; TIL – tumor–infiltrating lymphocytes; Treg – regulatory T cells; VEGF – vascular endothelial growth factor

The effect of *BRAF* mutations on proliferation and survival of affected cells is the most recognized and widely investigated outcome. However, some of the multifaceted additional impacts that this genetic aberration has upon signaling pathways in both the mutated cells themselves as well as on the surrounding, non-mutated cells within the tumor microenvironment are important as well (Fig. 1A). Oncogenic-induced senescence through upregulation of p16 expression has been primarily seen in RAS-mutated cells or its downstream effectors, RAF and MEK [18–21]. This senescence leads to growth arrest and a low proliferation rate in *BRAF-*mutated cells, an outcome that would seem to be contrary to achieving the all-important pro-proliferative and pro-survival goal of the *BRAF* mutation. Another function of p16 and other cyclin-dependent kinase inhibitors, e.g., p19 and p21, is to induce autophagy that is in an active and reciprocal relation with the induction of senescence [22]. Autophagy is a fundamental cellular process that eliminates molecules and sub-cellular elements via lysosomes to promote various activities, such as homeostasis, development, and differentiation [23]. Senescent cells are also characterized by several secreted factors, such as insulin-like growth factor binding protein-7 and senescence-associated secretory phenotype inflammasomes that have an impact upon tumor microenvironment (TME) cells [19, 20, 24]. Concurrently, fibroblasts undergo senescence (expression of p16) or apoptosis (expression of caspase 3), and some of them undergo transition into myofibroblasts. These senescence-related secreting factors also have an effect upon the type of inflammatory response, which is usually the immune suppressive type [25]. The main effects of BRAFi on the mutated cells consist of further inhibition of cell proliferation and survival, reversing the cytokine/chemokine profile from immune-suppressor to pro-inflammatory, and inducing cell death (Fig. 1B).

Initial microscopic examination of the post-treated residual ameloblastomas revealed that tumors had undergone remarkable architectural and cellular changes [15]. Interestingly, most of the reports on the results following the use of BRAFi (with/without MEKi) in the management of ameloblastomas have provided only minimal information on the post-surgery microscopic findings [7, 8, 10–13, 17]. An exception is that of Tan et al., [9] who were more specific and provided details on macroscopic cyst formation, microscopic degenerative changes, squamous differentiation, and neutrophil infiltration in the ameloblastoma lining epithelium at the luminal surface of the cystic structures.

The aim of the present study was to provide what we believe to be the first comprehensive description of changes in tumor morphology and selective molecular markers in either BRAFi- or combined BRAFi-MEKi-treated ameloblastomas. The importance of this study was discussed in light of the currently available knowledge of an evolving approach for treating ameloblastomas via targeted therapy and the need for a guiding tool for clinicians in outcome assessment associated with this approach.

## Methods

Between 2017 and 2023, the Department of Cranio-Maxillofacial Surgery, Chaim Sheba Medical Center, Tel Hashomer, Israel managed 14 patients diagnosed with *BRAF*-mutated ameloblastomas, whom completed targeted treatment and have undergone conservative definitive surgery that yielded sufficient tumor tissue for all the performed stains. Patients with large ameloblastomas that led to facial deformation were offered first line neoadjuvant therapy with BRAFi (with/without MEKi), as a jaw preservation approach (detailed protocol in supplementary information #1), instead of the standard of care surgery which would have comprised jaw resection with immediate reconstruction surgery via free vascularized tissue transfer. *BRAF* mutation status was performed using routine panels for standard next generation sequencing assays (NGS, Colon and Lung Cancer panel, AmpliSeq™, Thermo Fisher Scientific, Wilmington, DE, USA). Procedures included DNA extraction from paraffin sections containing areas with at least 90% tumor cells using Qiagen columns (Thermo Fisher Scientific, Wilmington, DE, USA). Ten nanograms of DNA (measured with Qubit^®^) were used for NGS. An amplicon library was generated for sequencing 1840 hotspot mutations in 22 genes including, BRAF, CTNNB1, KRAS, MAP2K1, NRAS, and TP53, using the “Colon and Lung Cancer panel” (Ampliseq™, Life Technologies). Raw data were analyzed using ion reporter software (Life Technologies). Each mutation was verified using Integrative Genome Viewer (IGV, Broad Institute). The *BRAF* mutation status alone was identified by the Biocartis Idylla system (Thermo Fisher Scientific) in three cases.

BRAFi/MEKi was provided through an expanded access compassionate program by Novartis Pharmaceuticals (Basel, Switzerland). The study was approved by the local IRB, SMC-9405-22 and performed in accordance with the Declaration of Helsinki.

The following data for all of the patients were retrieved from the institutional database of medical records: age, sex, diagnosis (conventional ameloblastomas, mural type unicystic ameloblastomas), frequency of *BRAF* mutation, treatment (BRAFi +/- MEKi), and treatment duration referring to time to maximal radiologic response with the greatest reduction in tumor size (nadir status), or earlier termination of the treatment due to either a lack of compliance, minimal radiological response, or adverse side effects [16]. Our definition for the microscopic response had been described in detail in our earlier report [16], and it was applied in the present study. In brief, a near-complete response was one that still displayed a few residual small, scattered clusters of ameloblastoma, while presence of changes in only part of the residual tumor alongside vital and morphologically unchanged tumor, was considered a partial response. We used a total of 7 parameters to evaluate the tumor response (especially for tumors with partial response), three of which were epithelial-related and four stroma-related, as detailed below.

### Histomorphologic Features and Estimation of Changes

The histopathologic features of conventional ameloblastomas and those of unicystic ameloblastomas are well recognized [1, 2, 26] and served as a reference for analyzing BRAFi/MEKi post-treatment changes. We examined changes of the epithelial component in terms architecture (classical peripheral palisades and central loose, stellate reticulum-like layering), its cellular modifications (appearance of eosinophilic cytoplasm, pyknotic nuclei, and intercellular loss of cohesion). In addition, we assessed the presence of squamous metaplasia/differentiation with keratin formation. The extent of these epithelial changes were graded as 0 = well-conserved tumor, 1 = focal changes affecting less than 50% of the tumor, and 3 = changes affecting 50% and more of the tumor. The rationale of a 0, 1 and 3 score (rather than 0, 1, 2) was aimed to highlight the features with the most remarkable changes and discriminate between low- and high-responder tumors.

The tumor stroma was assessed for changes of fibrosis, diffuse and granulomatous type of inflammatory reaction, new bone formation/woven bone trabeculae (within tumor stroma and not at its periphery). These stromal changes were scored as 0 = none, 1 = focal/minimal, and 3 = frequent.

We used alcian blue histochemical stain (Ventana BenchMark; Roche Tissue Diagnostics, Tucson, AZ, USA) in a qualitative manner to highlight the relatively loose/myxomatous areas. Masson tri-chrome (Ventana BenchMark) was used for those tumors with post-treatment extensive fibrosis.

Whenever feasible, we compared the features of the tumors before and after treatment using the same set of parameters and scores.

### Immunohistochemical Markers – Qualitative Estimation of Their Expression

Immunostaining with cytokeratin 19 was used qualitatively to emphasize the amount and extent of residual ameloblastoma in several cases. Immunostaining with p16 was also performed as it served as a marker for oncogenic activation of the mitogen-activated protein kinase (MAPK) pathway-induced cellular senescence [18, 27], and as its expression (nuclear and cytoplasmic) had been found to be associated with squamous differentiation in ameloblastomas [28]. We assessed apoptosis by means of caspase 3, using an antibody which identified its activated form as nuclear expression [29]. We highlighted macrophage infiltration in either a diffuse form or in a granulomatous pattern by immunostaining with CD68. Table 1 summarizes the technical details of the antibodies that were used.

**Table 1:** Antibodies used for the immunohistochemical study. All stains were performed using Ventana BenchMark Ultra immunostainer and protocols (Roche Tissue Diagnostics, Tucson, AZ, USA). Brown diaminobenzidine (DAB) staining was used to visualize positively stained cells

Primary Antibody	Targeted cell type	Manufacturer	Catalogue #/clone, Concentration	Positive control tissue/stained cellular compartment
p16	Senescent cells	CINtec, Ventana, Roche	9511/clone E6H4, prediluted	Cervical carcinoma/nuclear and cytoplasmic
Caspase 3	Apoptotic cells (32 kDa proenzyme and 17 kDa active form)	Abcam, Cambridge, UK	Ab4051/polyclonal, 1:200	Tonsil/nuclear
CD68	Macrophages	Dako, Glostrup, Denmark	M0876/KP1, 1:50	Tonsil/cytoplasm
CK19	Odontogenic epithelium/ ameloblastoma	Dako, Glostrup, Denmark	GA615/RCK108, 1:50	Appendix/cytoplasm
SATB2	Cells with osteoblastic phenotype	Cell Marque, Rocklin, CA, USA	384R/ EP281, 1:100	Appendix/nuclear
BRAF	Mutated ameloblastoma	Spring Biosciences, Pleasanton, CA, USA	E19294/VE1, 1:50	Melanoma/cytoplasm

### Statistical Analysis

The results were presented as the mean scores of the parameters for epithelium, stroma, and total tumor (i.e., the mean of epithelium + stroma). Differences in mean scores of the epithelium and stroma between tumors with partial response and tumors with a near-complete response were analyzed by Mann-Whitney test, and between before and after treatment were analyzed by paired T-test. Spearman’s correlation test was used to analyze correlations between the duration of treatment and the various microscopic mean scores (total, epithelium, stroma, and each of the individual histomorphologic parameters). Significance was set at *p* < 0.05. All analyses were performed using SPSS, version 28 (IBM, Chicago, IL, USA).

## Results

### Study Group

The study comprised 14 patients, 11 of whom were diagnosed as having mandibular tumors (near complete response, *n* = 3; partial response, *n* = 8) and three as having maxillary tumors (all partial response). Thirteen had the *BRAF*^*V600E*^ mutation and one with a maxillary tumor had a *BRAF-SND* fusion. Table 2 summarizes the patients’ clinical data, diagnosis (conventional, unicystic ameloblastoma - mural type), variation allele frequency of *BRAF*^*V600E*^, treatment modality (BRAFi/BRAFi + MEKi/MEKi), and duration of treatment. Variations in allele frequency of *BRAF*^*V600E*^ before the targeted treatment ranged between 2 and 57.87%, and none were related to the response to treatment. Statistical analysis was performed for the entire study group (*n* = 14), for the mandibular tumors (*n* = 11), for the mandibular tumors with partial response (*n* = 8), and for the mandibular tumors with near complete response (*n* = 3). Six cases were also compared for their pre- and post-treatment status.

**Table 2:** Clinical data, treatment regimen and microscopical scores per tumor, stroma and total (tumor + stroma)

		Mandible											Maxilla		
		Near Complete pathologic response			Partial pathologic response								Partial pathologic response		
Patient #		1*	2*	3*	4*	5*	6*	7*	8*	9*	10	11	12	13	14
Clinical features	Age at diagnosis (y)	15	16	15	10	21	83	13	63	19	37	42	63	72	43
	Diagnosis	U-M	U-M	U-M	U-M	U-M	AM	U-M	AM	U-M	U-M	AM	AM	AM	AM
	*VAF BRAF*^V600E^ (%)**	41.83	+	16	27.98	12.3	+	44.4	30	2	11.26	43	BRAF-SND fusion	57.87	+
	Therapeutic agent	Bi	Bi	Bi	Bi	Bi	Bi + Mi	Bi	Bi + Mi	Bi + Mi	Bi	Bi + Mi	Mi	Bi + Mi	Bi + Mi
	Duration of treatment (mo)	20	6	12	12	13	4	18	8	3	4.5	0.75	2	3	9
Epithelial scores	Architectural***	3	3	3	3	3	3	3	3	3	3	1	3	1	3
	Cellular****	3	1	3	1	3	1	3	3	3	3	3	3	1	3
	Squamous differentiation	1	0	-	1	3	1	0	1	0	1	1	3	3	3
	**Epithelial mean scores**	2.3	1.3	3	1.67	3	1.67	2	2.3	2	2.3	1.67	3	1.67	3
Stroma scores	Fibrosis	3	0	3	1	3	1	1	3	1	0	1	0	1	1
	Inflammation, diffuse	1	0	3	3	3	3	3	3	3	3	1	1	1	1
	Inflammation, granulomatous	3	0	3	1	3	0	3	0	1	1	0	0	1	0
	New bone within tumor stroma	0	0	1	0	0	0	0	1	1	1	1	0	1	0
	**Stroma mean scores**	1.75	0	2.5	1.25	2.0	1.0	1.75	1.75	1.25	1.25	0.75	0.25	1.0	0.5
**Total mean scores epithelium + stroma**		**2.025**	**0.65**	**2.75**	**1.46**	**2.5**	**1.335**	**1.875**	**2.025**	**1.625**	**1.775**	**1.21**	**1.625**	**1.335**	**1.75**

U-M – unicystic ameloblastoma, mural type; AM – ameloblastoma, conventional; VAF – variation allele frequency; Bi – BRAF inhibitor; Mi – MEK inhibitor; *Patient included in previous publication [16]; **performed using NGS as described in “Methods” section, while identification of only *BRAF* mutation (+) was performed by Biocartis Idylla system (Thermo Fisher Scientific); ***distorted layering, lost palisades; ****eosinophilic cytoplasm, pyknotic nuclei, dis-cohesion

### Histomorphologic and Molecular Changes

**Fig. 2:**
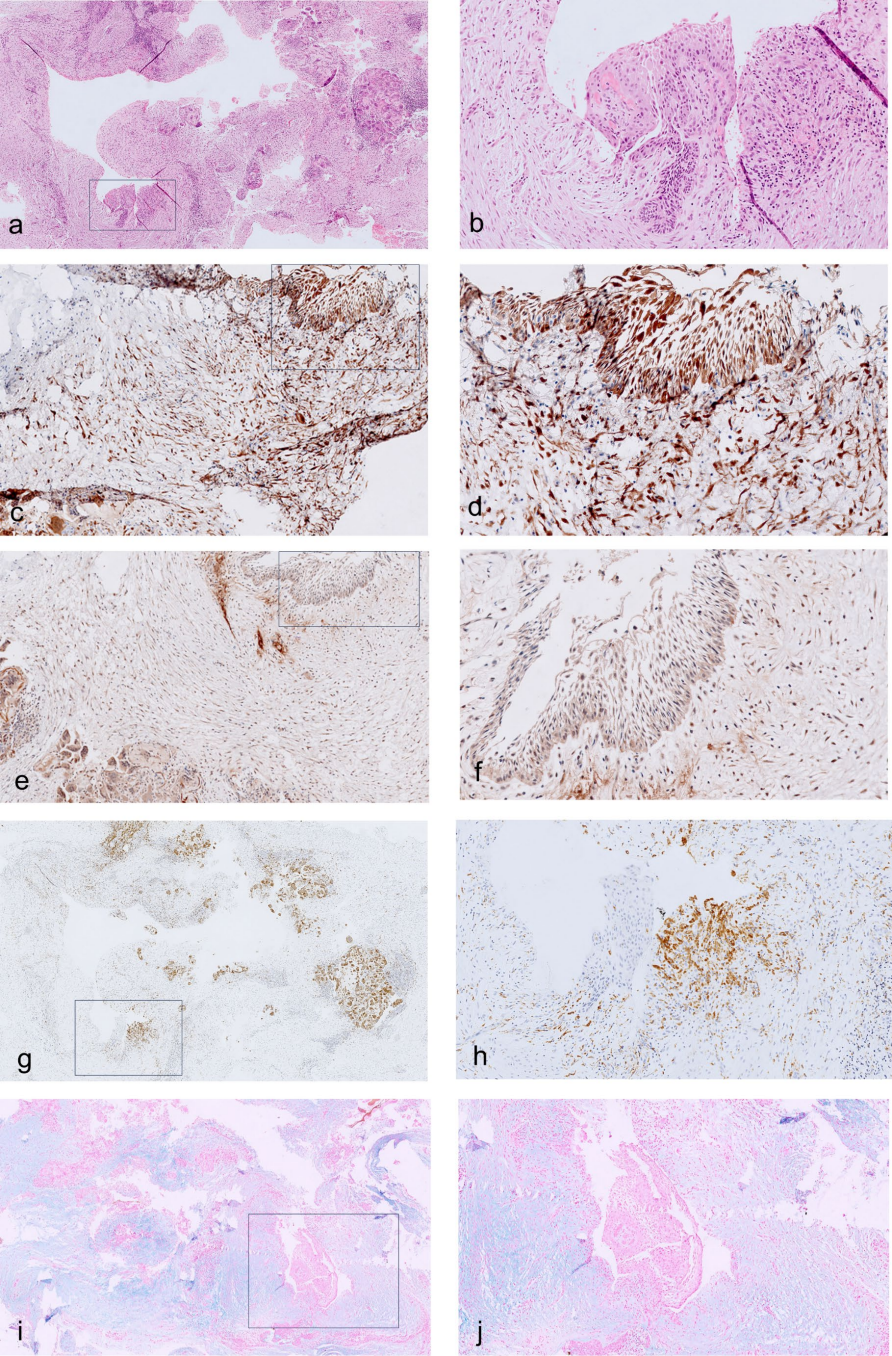
Photomicrographs of treated unicystic ameloblastoma, mural type, with a near complete response. (**a**) collapsed cystic structure, small residual lining fragment of ameloblastoma (rectangle); (**b**) residual ameloblastoma from “a” at higher magnification: non-specific features, vague basal palisades; (**c**) p16 immunostain: positive in residual ameloblastoma (rectangle) and stroma; (**d**) residual ameloblastoma from “c” at higher magnification; (**e**) caspase 3: positive in nuclei of stromal cells, residual ameloblastoma positive cytoplasm (rectangle); (**f**) caspase 3, rectangle from “e” at higher magnification: no nuclear staining in ameloblastoma; (**g**) CD68 immunostain: positive macrophages in granulomas associated with residual ameloblastoma (rectangle); (**h**) CD68 immunostain: positive scattered macrophages nearby focus of residual ameloblastoma; (**i**) alcian blue: myxoid stroma, weak intensity also nearby residual ameloblastoma (rectangle); (**j**) alcian blue: area in rectangle from “i” at a higher magnification

**Fig. 3:**
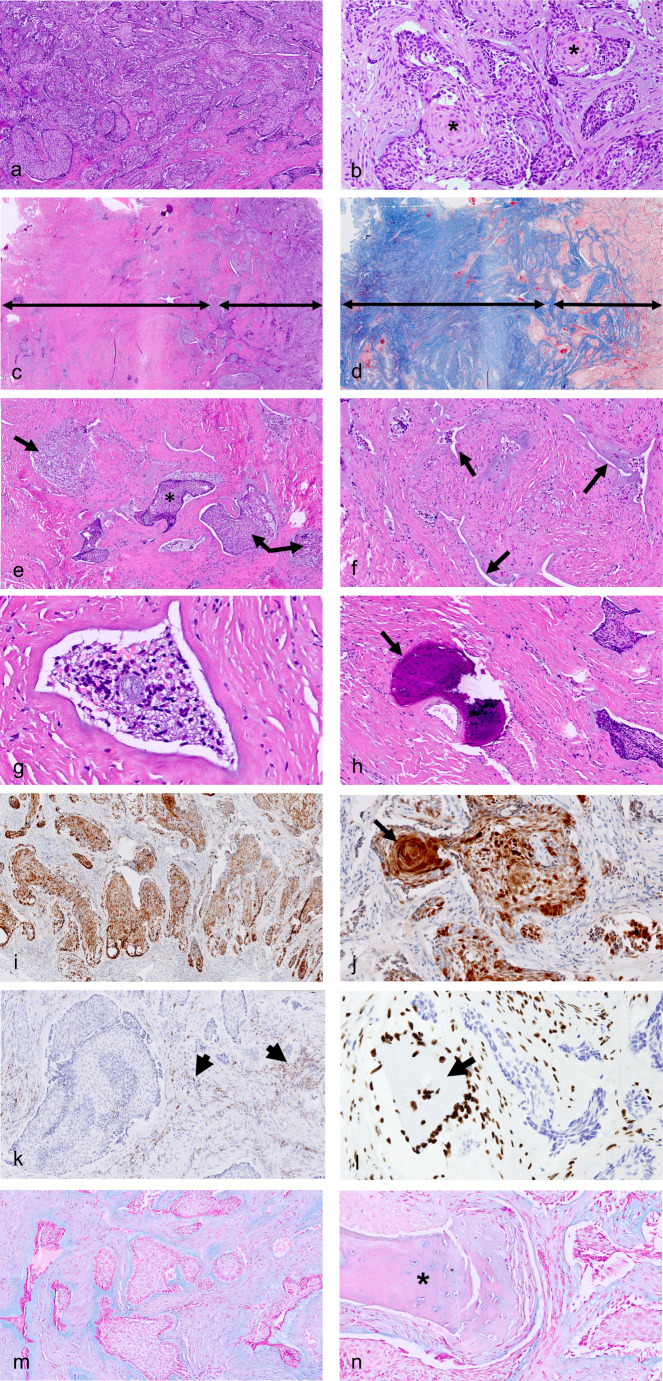
Photomicrographs of treated conventional ameloblastoma with partial response. (**a**) vital ameloblastoma; (**b**) ameloblastoma islands with acanthomatous/squamous changes in the center areas (asterisks); (**c**) right - vital ameloblastoma, left - highly dense collagenous/fibrotic stroma with non-vital ameloblastoma; (**d**) Masson tri-chrome stain, left: dense collagenous/fibrotic stroma, non-vital ameloblastoma; (**e**) collagenous/fibrotic area at higher magnification: islands of vital (asterisk) and non-vital ameloblastoma (arrows); (**f**) collagenous/fibrotic area at higher magnification: slit-like spaces containing tumor debris or entirely devoid of tumor (arrows), outlined by a bluish band of myxoid stroma; (**g**) - non-vital ameloblastoma; (**h**) - small new bone trabecula in fibrotic area (arrow); (**i**) – p16 immunostain - strongly positive tumor; (**j**) – p16 immunostain at higher magnification: keratin pearl formation (arrow); (**k**) - Caspase 3 immunostain: positive only in stromal cells (arrows); (**l**) - SATB2 immunostain: positive in association with a new bone small trabecula (arrow); (**m**). alcian blue stain: strongly stained band surrounding vital ameloblastoma; (**n**). alcian blue stain: weak intensity in area of new bone formation (asterisk)

**Fig. 4:**
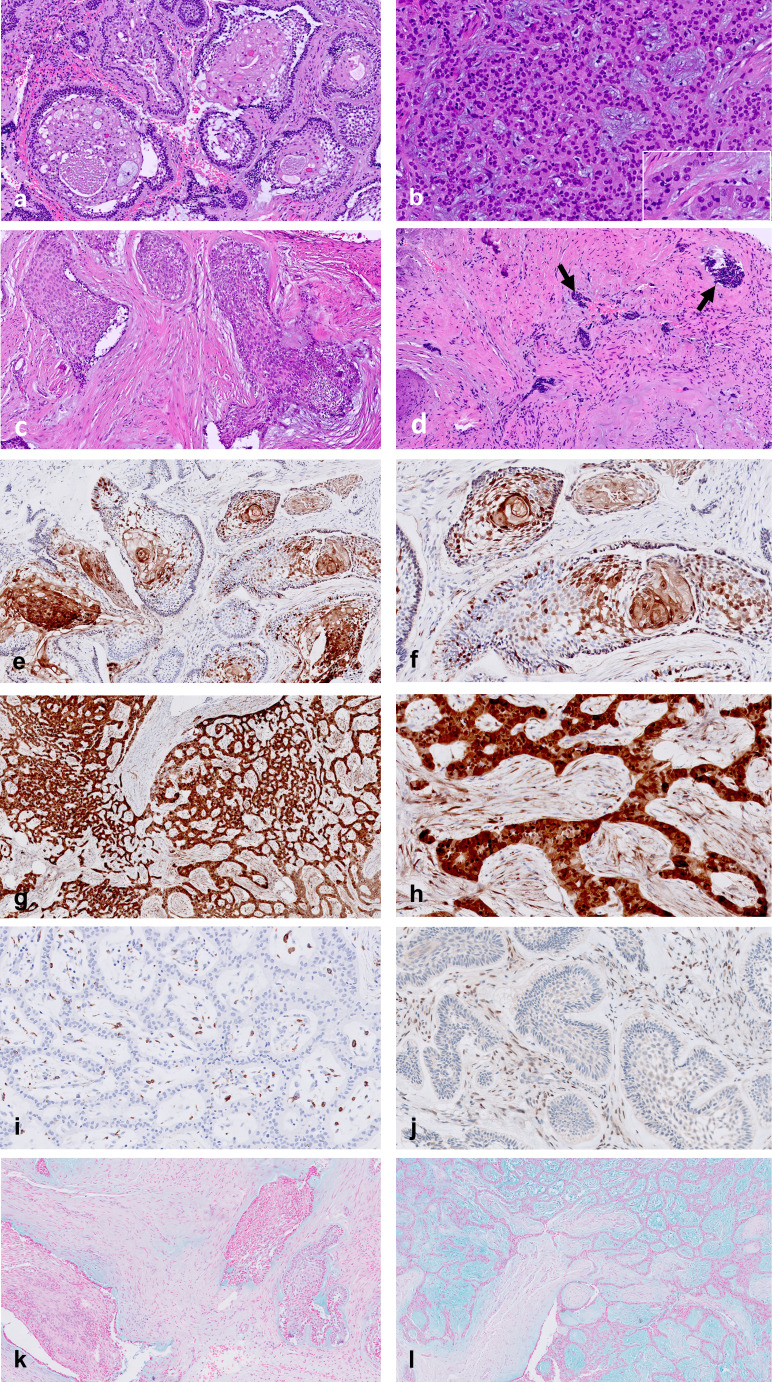
Photomicrographs of 3-week treated conventional ameloblastoma with partial response. (**a**) follicular area: partial loss of palisades, central areas with cystic changes, intercellular discohesion and squamous metaplasia; (**b**) plexiform area: columnar cells with extensive granular change indicating senescence (28); (**c**) follicular area: no palisading, squamous and spindle cell changes; (**d**) nests of distorted residual tumor (arrows); (**e**) p16 immunostain: highlights squamous change, including keratin pearl formation; (**f**) p16 immunostain, higher magnification from “e”: squamous change and keratin pearls; also positive stromal cells; (**g**) p16 immunostain: strongly positive in plexiform areas, abundant positive stromal cells; (**h**) p16 immunostain, higher magnification from “g”: strongly and uniformly positive in plexiform areas, abundant positive stromal cells; (**i**) CD68 immunostain: positive macrophages; (**j**) caspase 3: positive stromal cells; (**k**). alcian blue: strongly stained ameloblastoma-associated stroma, remaining stroma weakly stained; (**l**). alcian blue: strongly stained ameloblastoma-associated stroma in plexiform area, remaining stroma weakly/not stained

Considering the neoplastic epithelium, architectural and cellular histomorphologic changes were the most remarkable findings in post-treatment ameloblastomas, in both conventional and unicystic, and their extent appeared to increase with the duration of treatment (Figs. 2, 3 and 4). In tumors with partial response, in addition to areas of vital, regular ameloblastoma, there were parts of tumors that lost the characteristic peripheral palisading appearance. Furthermore, neoplastic epithelial cells of the solid or cystic tumors showed various combinations of eosinophilic cytoplasm with/without granular appearance, hyperchromatic nuclei. and inter-cellular dis-cohesion. The stellate reticulum-like areas within the central areas of solid epithelial structures occasionally showed full squamous differentiation, including formation of desmosomes and keratin pearls. The greatest effect of the treatment was noted when the epithelial tumor islands were severely deformed and distorted, with only some ruptured cellular and nuclear debris as the sole evidence for the previous existence of a tumor.

**Fig. 5:**
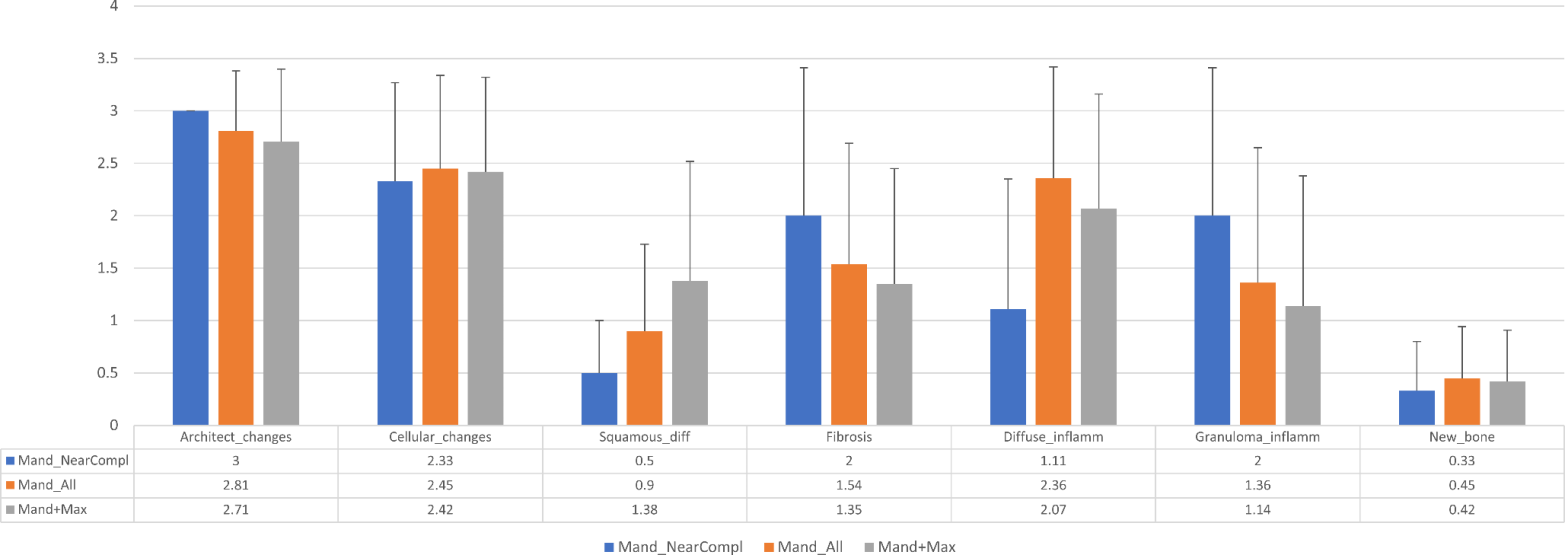
Mean scores of histomorphologic parameters in post-treated ameloblastomas. Abbreviations: Mand_NearCompl – mandibular tumors with near-complete response (*n* = 3); Mand_All – all treated mandibular tumors (*n* = 11); Mand + Max – all treated tumors, mandibular and maxillary (*n* = 14); Architect_changes – architectural changes; Squamous_diff – squamous differentiation; Diffuse_inflamm – diffuse inflammation; Granuloma_inflamm – granulomatous inflammation; New_bone – new bone formation

Considering stromal changes, the inflammatory reaction was the most noticeable finding in post-treated tumors. Varying degrees of scattered chronic inflammation with or without granulomas were also apparent. Granulomas seemed to be common in areas where the tumor was no longer identified as having being replaced by a fibrotic connective tissue, which was highlighted by Masson tri-chrome stain. The lumina of cystic tumors were filled with tumor debris usually accompanied by mostly chronic inflammatory cells, either scattered or as a granulomatous type of inflammation. Myxoid niches of stroma closely surrounded the tumoral epithelium or occupied more extensive areas – these were highlighted by alcian blue stain. We could identify small trabeculae of osteoid or woven bone surrounded by plump osteoblasts within the tumor stroma in several tumors. More substantial bone formation was occasionally observed at the periphery of the tumors. The total scores for each examined parameter, epithelium, and stroma together with their mean scores are shown in Table 2; Fig. 5.

Immunostaining revealed that the distribution of post-treatment expression of p16 varied in both the neoplastic epithelium as well as in the stroma, ranging between strong to no reaction, even within the same tumor. The expression of p16 frequently highlighted the areas with squamous differentiation but was not limited to these areas. p16 also highlighted cells with a spindle-shaped morphology within the stroma. Expression of caspase 3 in the neoplastic epithelium was almost absent, in contrast to many of the stromal cells being positive.

### Duration of Treatment and Epithelium and Stroma Scores

Table 3 summarizes the mean epithelium and stroma scores and the combined (epithelium + stroma) scores for all 14 examined samples. Specifically, the mandibular tumors were subdivided further into those with partial (*n* = 8) and near-complete response (*n* = 3). The mean epithelial score of all 14 tumors in combination (2.11 *±* 0.57) was higher than that of the mean stroma score (1.25 *±* 0.7) (*p* = 0.003). This pattern was similar to the scores for all mandibular tumors (epithelium = 2.12 *±* 0.51 and stroma = 1.433 *±* 0.66; *p* = 0.035). Tumors with partial response did not differ significantly from those with near-complete response with regard to mean total epithelial + stroma, epithelial, and stroma scores (*p* = 0.65, *p* = 0.55, and *p* = 0.27, respectively).

**Table 3:** Mean scores of the epithelial parameters, stromal parameters and total (epithelium and stroma) parameters for all 14 examined tumors, for the 11 mandibular tumors, for mandibular tumors with partial response (*n* = 8) and tumors with near complete response (*n* = 3)

	Mean duration of treatment (mo)	Mean total (epithelial + stroma) score	Mean epithelial score	Mean stroma score
All tumors (*n* = 14)	8.23 *±* 6.03	1.*73 +* 0.52	2.11 *±* 0.57^1^	1.25 *±* 0.7^1^
Mandibular tumors (*n* = 11)	9.2 *±* 6.0	1.*77 +* 0.57	2.12 *±* 0.51^2^	1.43 *±* 0.66^2^
Mandibular tumors with partial response (*n* = 8)	7.9 *±* 5.55	1.76 *±* 0.42^5^	2.08 *±* 0.43^3,6^	1.43 *±* 0.44^3,7^
Mandibular tumors with near complete response (*n* = 3)	12.67 *±* 5.73	1.*81 +* 0.86^5^	2.22 *±* 0.68^4,6^	1.4*1 +* 1.04^4,7^

^1^*p*=0.003; ^2^*p*=0.035; ^3^*p*=0.031; ^4^*p*=0.31; ^5^*p*=0.65; ^6^*p*=0.27; ^7^*p*=0.55

The mean epithelial score of the total cohort (*n* = 14) did not correlate with treatment duration (*r* = 0.3, *p* = 0.28), whereas the mean total and stroma scores correlated positively with treatment duration (*r* = 0.64, *p* = 0.013 and *r* = 0.66, *p* = 0.01, respectively). Architectural changes, fibrosis, and granulomatous type of inflammation also correlated significantly with treatment duration (*r* = 0.533, *p* = 0.05; *r* = 0.562, *p* = 0.037; *r* = 0.672, *p* = 0.008, respectively).

Treatment duration of mandibular tumors (*n* = 11) correlated with the stroma score (*r* = 0.66, *p* = 0.025) and the total (*r* = 0.64, *p* = 0.03) but not with the epithelial score (*r* = 0.47, *p* = 0.14). Among all of the histomorphologic parameters examined in this study, the formation of a granulomatous type of inflammation emerged as being the only one significantly associated with the duration of treatment (*r* = 0.7602, *p* = 0.007).

For each patient, the total score of the epithelium was always higher than that of the total score of the stroma, irrespective of treatment duration. However as treatment was longer, the total score that the stroma achieved was higher and closer to that of the epithelium (Supplemental Information #2).

### Epithelium, Stroma and Total Tumor Scores, Before *Versus* After Treatment

Table 4 summarizes the mean epithelial and stromal scores of all examined parameters in the six cases, for which tissue was available for comparisons. Significant differences were found regarding the epithelial architectural and cellular changes and stromal fibrosis and granulomatous type of inflammation. The overall after treatment epithelial and stromal scores were significantly higher than those before treatment (2.27 *±* 0.4 and 0.64 *±* 0.063, *p* = 0.005, and 1.66 *±* 0.34 and 0.7*5 +* 0.28, respectively, *p* = 0.003).

**Table 4:** Mean scores of epithelial and stromal parameters, separated and combined, before versus after treatment

	Epithelium			Stroma			
	Architectural changes	Cellular changes	Squamous differentiation	Fibrosis	Inflammation, diffuse	Inflammation, granulomatous	New bone formation in tumor stroma
Before treatment	0	1	1.33 *±* 0.74	0	1.43 *±* 1.05	0	0.14 + 0.35
After treatment	3	2.71 *±* 0.7	1.17 *±* 0.89	1.57 *±* 1.29	2.43 *±* 0.9	1.57 *±* 1.29	0.43 + 0.49
*P* value	*p* < 0.001	*p* < 0.001	*p* = 0.363	*p* = 0.025	*p* = 0.086	*p* = 0.025	*p* = 0.172

## Discussion

The present study aimed to provide a novel description and measurable assessment of the histomorphologic modifications and selective molecular changes in *BRAF*-mutated, target-treated ameloblastomas. We separately scored the neoplastic epithelium and surrounding stroma and correlated these changes with treatment duration. Although preliminary, the results revealed tumor-specific changes of the neoplastic epithelium and its stroma that have occurred in what seems to be a predictable time-related manner and were not necessarily related to the type of treatment (single/combination, dose). Specifically, the neoplastic epithelium appeared to have responded to treatment first and rapidly, after which the stromal response (inflammation and fibrosis) evolved and grew larger during treatment, thus enhancing the therapeutic effect of the inhibitors – a response process that usually has developed over a prolonged period of time (weeks-to-months). Our results may raise the possibility that treated ameloblastomas carry the potential for (near) complete microscopic response but that its achievement depends upon complex and individual tumor- and patient/treatment-related factors, such as age, comorbidities, bone metabolism rate (young *versus* elder patients), compliance with treatment, and adverse side effects. The suggested scoring system may add insights especially for tumors with partial response in terms of assessing which compartment of the tumor has not responded as expected and aid the clinical team for planning additional adjuvant treatment options aimed at jaw preservation.

BRAFi-related histomorphologic changes have thus far been reported in malignant tumors, and these alterations seem to be tumor type-related. For example, post-treatment histomorphologic changes in melanomas are comprised of small tumor cells with eosinophilic cytoplasm and hyperchromatic and pleomorphic nuclei [18], suggesting a process of apoptosis molecularly supported by caspase 3 (a marker for apoptosis) in only a negligible fraction of tumor cells [30]. Post-treatment extensive tumor necrosis was reported on other BRAFi-treated tumors, such as anaplastic thyroid carcinoma [31, 32], and as usually being anticipated in tumors with high proliferation rates [33]. While malignant tumors carry several critical mutations that lead to high proliferation when in combination [15], ameloblastomas are heavily reliant upon the driver *BRAF* mutation [7–9, 34] that is associated with low proliferation rates. The current series comprised patients that had been treated for different periods of time, therefore providing us with the opportunity to examine microscopic changes in a time-wise manner. We found significant changes in epithelial architectural and cellular epithelial in post-treatment tumors compared to the counterpart pre-treatment status. These post-treatment findings showed unique and consistent tumor-related changes, which displayed a similar trend of changes for all treatment durations. These changes could not be attributed to apoptosis, since caspase 3 was almost entirely absent. The absence of necrosis can be explained by a low proliferation rate conferred by *BRAF* mutation-induced senescence, as reflected by strong and diffuse expression of p16 [35–39]. In terms of treatment, this low proliferation rate is a feasible explanation for the expected slow clinical response of ameloblastomas to BRAFi [8, 9, 17, 34] extending over several weeks and months, unlike malignant tumors that usually show a rapid response. In addition, treatment regimen (single/combination, dose) is not expected to change the fairly predictable time-related pattern of response in ameloblastoma.

Squamous changes were a common feature in post-treatment ameloblastomas. This finding has been similarly reported in the literature [9, 17], with the suggestion that treatment with BRAFi could have directed the tumor towards a more mature phenotype [9]. Interestingly, we observed an overlap between p16 expression and areas showing squamous differentiation, as had also been observed by others [28]. Although BRAFi agents themselves have the potential to induce senescence (upregulated p16 expression) in targeted ameloblastoma cells [40, 41], their contribution was not found to be significant when pre- and post-treatment tumors were compared. However, the joint senescence impact of the *BRAF* mutation and BRAFi in the setting of ameloblastoma is probably followed by an effect of autophagy, which is likely associated with further squamous differentiation of cells. Autophagy has been recently shown to occur in ameloblastomas [42], raising the possibility of a sequence of events comprised of *BRAF* mutation/BRAFi leading to senescence (p16 expression), autophagy, and squamous differentiation. An impact on the adjacent TME cells through BRAFi-induced senescence in the neoplastic epithelium, causing TME cells to undergo senescence (expression of p16) and apoptosis (caspase 3) [19, 20, 24], occurred in the treated tumors. We can suggest that the additional impact of BRAFi to that of *BRAF* mutations in this aspect was in regard to senescent fibroblasts that underwent transition into myofibroblasts, ultimately leading to significant fibrosis in the treated tumors.

The extracellular matrix of ameloblastomas had been revealed to be determined by the neoplastic epithelium that expresses factors that positively control osteoclastogenesis, such as a receptor activator for nuclear factor κappa B ligand (RANKL) and interleukin (IL)-6, on the one hand, while also expressing factors, such as secreted-frizzled-related protein-2 that suppresses osteoblasts, on the other hand [43]. This creates a TME that blocks new bone formation and encourages bone absorption. That study also found that these effects were intensified in ameloblastomas with myxoid stroma. It has been recently reported that a myxoid type of matrix may represent a state of senescence with a distinct inflammatory signature (i.e., rich in macrophages and cytotoxic T cells) as well as a rich vasculature [44]. Senescence damages bone remodeling by impairing bone formation and osteoblast progenitor cell function, thus promoting osteoclastogenesis [45, 46]. We found extensive myxoid matrix in many of BRAFi-treated ameloblastomas, as well as abundant stromal expression of p16. Collectively, we can assume that a myxoid stroma rich in senescent cells might maintain a pro-osteoclastic environment in ameloblastomas and thus could preclude bone regeneration. By eliminating the neoplastic epithelium, BRAFi reduces one pro-osteoclast-promoting factor, however, these are preliminary findings that emphasize the complexity of interactions and necessitate further research on the nature of the extracellular matrix (myxoid *versus* fibrotic) in the response to targeted therapy.

One of the most important known effects of BRAFi is its impact on the inflammatory response by creating an anti-tumor environment by recruiting tumor-infiltrating lymphocytes and cytotoxic lymphocytes and down-regulating immune suppressor factors (Fig. 1B) [25]. As a result, tumor cells undergo pyroptosis, which is an inflammation-associated cell death [25], rather than apoptosis. Our results are in line with this: we found a marked inflammatory reaction that targeted and destroyed the neoplastic epithelium. Lack of expression of caspase 3 indicated the absence of apoptosis, thus the final word on cell death pathways in BRAFi-treated ameloblastomas awaits further research. A macrophage-rich infiltration including a granulomatous type of inflammatory reaction, which we found to be related to the duration of treatment, seemed to represent the final step in the tumor destruction and debris clearance following BRAFi [18, 47]. The granulomatous inflammation constituted a significant change in post-treated tumors and it is in conform with documented immune-related pathologic response criteria in other target-treated tumors [48, 49].

### When Pathology Meets the Clinics and Vice Versa

Collectively, the above-described morphological and molecular changes in BRAFi-treated ameloblastoma were specific for this tumor and likely dependent upon built-in features. We can now assume that these tumors, especially those in the mandible, followed a predictable pattern of behavior with emphasis upon a relatively slow reaction to treatment, irrespective of treatment regimen, a feature which should be taken into consideration by the clinicians. Other potential factors that could affect the time and extent of response are patient-related, as already mentioned, which should be discussed by a multidisciplinary team.

The case for a conservative approach for ameloblastomas *versus* resection with safety margins of 1.5 cm beyond the radiological margins has already and eloquently been debated throughout the pre-BRAFi era [50]. The stated reasons included the disproportionate number of these tumors occurring in young patients, the subsequent impact on quality of life subsequent to a radical operation (all patients), and a 15–25% rate of recurrence [51, 52]. According to our findings, cases with a near-complete pathological response retained minimal tumor residue that could be successfully removed conservatively, highlighting the need for a meticulous follow-up protocol. In regard to those tumors with partial response, analyzing microscopic findings according to the series of suggested parameters, should aid the treating team in understanding which tumor component has responded or failed to respond, and serve as a basis for decision taking toward subsequent steps in adjuvant treatment planning (e.g., follow up, conservative surgery, modifications/changes in targeted treatment regimen, combinations of these approaches). This is especially pertinent to mandibular ameloblastomas, even those with a large recurrent lesion, for which BRAFi treatment could be reinstated with a substantial likelihood of success [13], since these tumors are driven by one mutation with almost no other allied mutations and a low risk for the tumor to escape from targeted therapy.

### Study Limitations

One limitation of this study is the small number of cases for comparing the morphological and molecular features between the original and post-treated tumors in all patients. Since some of the patients were referred to us from other national and international medical centers, the paraffin blocks of the original tumors were not always available for molecular evaluation. Histomorphologic parameters and molecular markers examined in this initial study were selected on the basis of their being connected to known effector pathways of the *BRAF* mutation and impacts elicited by BRAFi, without previous knowledge of what to expect. It is to be assumed that comparing a higher number of pre- and post-treatment specimens will pave the way to update the presented panel of parameters and focus on those with immediate clinical application. Collaborative international studies with larger numbers of targeted-treated ameloblastomas are likely to enable the establishment of clinical, histopathologic, and molecular databases, which will ultimately aid in creating new guidelines for the treatment of this tumor.

## Conclusion

Treatment of ameloblastoma with targeted therapy is predictable and time related, enabling jaw preservation treatment strategy. Unlike malignant soft tissue tumors, when treating intra bony ameloblastomas, several months of treatment duration is expected due to the tumor’s slow clinical response and the associated bone metabolism. Time-wise, the reaction of the neoplastic epithelium is first and rapid, while the stromal response (inflammation and fibrosis) evolves and increases during treatment, the entire process expanding over weeks-to-months. Post surgical pathological evalutaion of the tumor tissue is essential as it can highlight which tumor components have responded or failed to respond. This should serve as the basis for the clinical decision on the need for adjuvant treatment, with a prime aim of jaw preservation, prevention of reccurent disease and minimizing sequelae.

## Electronic Supplementary Material

Below is the link to the electronic supplementary material.

Supplementary Material 1: Supplementary Information #1 (PDF). Protocol for ameloblastoma upfront targeted therapy implemented at Sheba Medical Center, May 2023

Supplementary Material 2: Supplementary Information #2 (PDF). Mean histomorphologic scores of tumor and stroma as a factor of duration of targeted treatment per patient

## Data Availability

All data generated and analyzed during this study are included in this manuscript.

## References

[1] Vered M, Adebiyi KE, Heikinheimo K (2022) Ameloblastoma. In: Muller S, Odell EW, Tilakaratne WM (eds) WHO classification of head and neck tumours. Odontogenic and maxillofacial bone tumours. IARC, Lyon. in press

[2] Neville BW, Damm DD, Allen CM, Chi AC (2023) Oral and maxillofacial pathology. Elsevier, St. Louis

[3] Borrelli MR, Hu MS, Longaker MT, Lorenz HP (2020) Tissue engineering and regenerative medicine in craniofacial reconstruction and facial aesthetics. J Craniofac Surg 31:15–27. 10.1097/SCS.0000000000005840 31369496 10.1097/SCS.0000000000005840 PMC7155741

[4] Brown NA, Rolland D, McHugh JB, Weigelin HC, Zhao L, Lim MS, Lim MS, Elenitoba-Johnson KS, Betz BL (2014) Activating FGFR2-RAS-BRAF mutations in ameloblastoma. Clin Cancer Res 20:5517–5526. 10.1158/1078-0432.CCR-14-1069 24993163 10.1158/1078-0432.CCR-14-1069

[5] Kurppa KJ, Catón J, Morgan PR, Ristimäki A, Ruhin B, Kellokoski J, Elenius K, Heikinheimo K (2014) High frequency of BRAF V600E mutations in ameloblastoma. J Pathol 232:492–498. 10.1002/path.4317 24374844 10.1002/path.4317 PMC4255689

[6] Sweeney RT, McClary AC, Myers et al (2014) Identification of recurrent SMO and BRAF mutations in ameloblastomas. Nat Genet 46:722–725. 10.1038/ng.2986 24859340 10.1038/ng.2986 PMC4418232

[7] Kaye FJ, Ivey AM, Drane WE, Mendenhall WM, Allan RW (2014) Clinical and radiographic response with combined BRAF-targeted therapy in stage 4 ameloblastoma. J Natl Cancer Inst 107:378. 10.1093/jnci/dju378 25475564 10.1093/jnci/dju378

[8] Faden DL, Algazi A Durable treatment of ameloblastoma with single agent BRAFi re: clinical and radiographic response with combined BRAF-targeted therapy in stage 4 ameloblastoma (2016). J Natl Cancer Inst 109:djw190. 10.1093/jnci/djw190 10.1093/jnci/djw190 27671684

[9] Tan S, Pollack JR, Kaplan MJ, Colevas AD, West RB (2016) BRAF inhibitor treatment of primary BRAF-mutant ameloblastoma with pathologic assessment of response. Oral Surg Oral Med Oral Pathol Oral Radiol 122:e5–7. 10.1016/j.oooo.2015.12.016 27209484 10.1016/j.oooo.2015.12.016

[10] Fernandes GS, Girardi DM, Bernardes JPG, Fonseca FP, Fregnani ER (2018) Clinical benefit and radiological response with BRAF inhibitor in a patient with recurrent ameloblastoma harboring V600E mutation. BMC Cancer 18:887. 10.1186/s12885-018-4802-y 30208863 10.1186/s12885-018-4802-y PMC6134697

[11] Brunet M, Khalifa E, Italiano A (2019) Enabling precision medicine for rare head and neck tumors: the example of BRAF/MEK targeting in patients with metastatic ameloblastoma. Front Oncol 9:1204. 10.3389/fonc.2019.0120 31781502 10.3389/fonc.2019.01204 PMC6861385

[12] Broudic-Guibert M, Blay JY, Vazquez L, Evrard A, Karanian M, Taïeb S, Hoog-Labouret N, Oukhatar CMA, Boustany-Grenier R, Arnaud A (2019) Persistent response to vemurafenib in metastatic ameloblastoma with BRAF mutation: a case report. J Med Case Rep 13:245. 10.1186/s13256-019-2140-6 31340860 10.1186/s13256-019-2140-6 PMC6657072

[13] Abramson Z, Dayton OL, Drane WE, Mendenhall WM, Kaye FJ (2022) Managing stage 4 ameloblastoma with dual BRAF/MEK inhibition: a case report with 8-year clinical follow-up. Oral Oncol 128:105854. 10.1016/j.oraloncology.2022.105854 35447565 10.1016/j.oraloncology.2022.105854

[14] Corbett K, Ruether D, Seiden-Long I, Kline G (2024) Resolution of PTHrP-mediated hypercalcemia following treatment with dual BRAF/MEK inhibition for BRAFV600E-positive metastatic ameloblastoma. Calcif Tissue Int 114:444–449. 10.1007/s00223-023-01177-x 38252285 10.1007/s00223-023-01177-x

[15] Hirschhorn A, Campino GA, Vered M, Greenberg G, Yacobi R, Yahalom R, Barshack I, Toren A, Amariglio N, Rechavi G (2021) Upfront rational therapy in BRAF V600E mutated pediatric ameloblastoma promotes ad integrum mandibular regeneration. J Tissue Eng Regen Med 15:1155–1161. 10.1002/term.3254 34599642 10.1002/term.3254

[16] Grynberg S, Vered M, Shapira-Frommer, Asher N, Ben-Betzalel G, Stoff R, Steinberg Y, Amariglio N, Greenberg G, Barshack I, Toren A, Yahalom R, Schachter J, Rechavi G, Hirschhorn A, Abebe Campino G (2024) Neoadjuvant BRAF targeted therapy for ameloblastoma of the mandible: an organ preservation approach. J Natl Cancer Inst 116:539–546. 10.1093/jnci/djad232 37966914 10.1093/jnci/djad232

[17] Daws S, Chaiyasate K, Lehal A (2021) Treatment of a BRAF V600E positive ameloblastoma in a pediatric patient with MEK inhibitor monotherapy. Face 2:179–182. 10.1177/27325016211005126

[18] Curry JL, Falchook GS, Hwu WJ, Torres-Cabala CA, Duvic M, Tetzlaff MT, Prieto VG (2013) Changes in tumor morphology and cyclin-dependent kinase inhibitor expression in metastatic melanoma treated with selective second-generation BRAF inhibitor. Am J Dermatopathol 35:125–128. 10.1097/DAD.0b013e318263f232 22878367 10.1097/DAD.0b013e318263f232

[19] Safwan-Zaiter H, Wagner N, Wagner KD (2022) P16INK4A - more than a senescence marker. Life (Basel) 12:1332. 10.3390/life12091332 36143369 10.3390/life12091332 PMC9501954

[20] Wajapeyee N, Serra RW, Zhu X, Mahalingam M, Green MR (2008) Oncogenic BRAF induces senescence and apoptosis through pathways mediated by the secreted protein IGFBP7. Cell 132:363–374. 10.1016/j.humpath.2009.12.002 18267069 10.1016/j.cell.2007.12.032 PMC2266096

[21] Azazmeh N, Assouline B, Winter E, Ruppo S, Nevo Y, Maly A et al (2020) Chronic expression of p16INK4a in the epidermis induces wnt-mediated hyperplasia and promotes tumor initiation. Nat Commun 11:2711. 10.1038/s41467-020-16475-3 32483135 10.1038/s41467-020-16475-3 PMC7264228

[22] Capparelli C, Chiavarina B, Whitaker-Menezes D, Pestell TG, Pestell RG, Hulit J, Meir K, Witkiewicz AK, Cohen J, Rizou SV, Pikarsky E, Luxenburg C, Gorgoulis VG, Ben-Porath I (2012) CDK inhibitors (p16/p19/p21) induce senescence and autophagy in cancer-associated fibroblasts, fueling tumor growth via paracrine interactions, without an increase in neo-angiogenesis. Cell Cycle 11:3599–3610. 10.1038/s41467-020-16475-3 22935696 10.4161/cc.21884 PMC3478311

[23] Aman Y, Schmauck-Medina T, Hansen M, Morimoto RI, Simon AK, Bjedov I, Palikaras K, Simonsen A, Johansen T, Tavernarakis N, Rubinsztein DC, Partridge L, Kroemer G, Labbadia J, Fang EF (2021) Autophagy in healthy aging and disease. Nat Aging 1:634–650. 10.1038/s43587-021-00098-4 34901876 10.1038/s43587-021-00098-4 PMC8659158

[24] Andrade AM, Sun M, Gasek NS, Hargis GR, Sharafieh R, Xu M (2022) Role of senescent cells in cutaneous wound healing. Biology (Basel) 11:1731. 10.3390/biology11121731 36552241 10.3390/biology11121731 PMC9775319

[25] Lelliott EJ, McArthur GA, Oliaro J, Sheppard KE (2021) Immunomodulatory effects of BRAF, MEK, and CDK4/6 inhibitors: implications for combining targeted therapy and immune checkpoint blockade for the treatment of melanoma. Front Immunol 12:661737. 10.3389/fimmu.2021.661737 34025662 10.3389/fimmu.2021.661737 PMC8137893

[26] Sciubba JS, Fantasia JE, Kahn LB (2001) Atlas of tumor pathology. Tumors and cysts of the jaws. 3rd series, Fascicle 29. Armed Forces Institute of Pathology, Washington, D.C

[27] Bennett DC, Medrano EE (2002) Molecular regulation of melanocyte senescence. Pigment Cell Res 15:242–250. 10.1034/j.1600-0749.2002.02036.x 12100489 10.1034/j.1600-0749.2002.02036.x

[28] Morice A, Neiva C, Fabre M, Spina P, Jouenne F, Galliani E, Vazquez MP, Picard (2020) Conservative management is effective in unicystic ameloblastoma occurring from the neonatal period: a case report and a literature review. Oral Surg Oral Med Oral Pathol Oral Radiol 129:e234–e242. 10.1016/j.oooo.2019.08.009 31562035 10.1016/j.oooo.2019.08.009

[29] Kamada S, Kikkawa U, Tsujimoto Y, Hunter T (2005) Nuclear translocation of caspase-3 is dependent on its proteolytic activation and recognition of a substrate-like protein(s). J Biol Chem 280:857–860. 10.1074/jbc.C400538200 15569692 10.1074/jbc.C400538200

[30] Johnson AS, Crandall H, Dahlman K, Kelley MC (2015) Preliminary results from a prospective trial of preoperative combined BRAF and MEK-targeted therapy in advanced BRAF mutation-positive melanoma. J Am Coll Surg 220:581–593e1. 10.1016/j.jamcollsurg.2014.12.057 25797743 10.1016/j.jamcollsurg.2014.12.057

[31] McCrary HC, Aoki J, Huang Y, Chadwick B, Kerrigan K, Witt B, Hunt JP, Abraham D (2022) Mutation based approaches to the treatment of anaplastic thyroid cancer. Clin Endocrinol (Oxf) 96:734–742. 10.1111/cen.14679 35067961 10.1111/cen.14679

[32] Maurer E, Eilsberger F, Wächter S, Riera Knorrenschild J, Pehl A, Holzer K, Neubauer A, Luster M, Bartsch DK (2023) Mutation-based, short-term neoadjuvant treatment allows resectability in stage IVB and C anaplastic thyroid cancer. Eur Arch Otorhinolaryngol 280:1509–1518. 10.1007/s00405-023-07827-y 36637521 10.1007/s00405-023-07827-y PMC9899736

[33] Rasbridge SA, Gillett CE, Seymour AM, Patel K, Richards MA, Rubens RD, Millis RR (1994) The effects of chemotherapy on morphology, cellular proliferation, apoptosis and oncoprotein expression in primary breast carcinoma. Br J Cancer 70:335–341. 10.1038/bjc.1994.303 7914426 10.1038/bjc.1994.303 PMC2033492

[34] Gates JC, Clark AP, Cherkas E, Shreenivas AV, Kraus D, Danzinger N, Huang RSP, Johnson J, Ross JS (2023) Genomic profiling and precision medicine in complex ameloblastoma. Head Neck 45:816–826. 10.1002/hed.27294 36645099 10.1002/hed.27294

[35] Kumamoto H, Kimi K, Ooya K (2001) Detection of cell cycle-related factors in ameloblastomas. J Oral Pathol Med 30:309–315. 10.1034/j.1600-0714.2001.300509.x 11334468 10.1034/j.1600-0714.2001.300509.x

[36] Tao Q, Lv B, Qiao B, Zheng CQ, Chen ZF (2009) Immortalization of ameloblastoma cells via reactivation of telomerase function: phenotypic and molecular characteristics. Oral Oncol 45:e239–244. 10.1016/j.oraloncology.2009.08.007 19833545 10.1016/j.oraloncology.2009.08.007

[37] Moreira PR, Guimarães MM, Gomes CC, Diniz MG, Brito JA, de Castro WH, Gomez RS (2009) Methylation frequencies of cell-cycle associated genes in epithelial odontogenic tumours. Arch Oral Biol 54:893–897. 10.1016/j.archoralbio.2009.07.006 19679296 10.1016/j.archoralbio.2009.07.006

[38] Singh T, Chandu A, Clement J, Angel C (2017) Immunohistochemistry of five molecular markers for typing and management of ameloblastomas: a retrospective analysis of 40 cases. J Maxillofac Oral Surg 16:65–70. 10.1007/s12663-016-0923-5 10.1007/s12663-016-0923-5 PMC5328870 28286387

[39] Olimid DA, Florescu AM, Cernea D, Georgescu CC, Mărgăritescu C, Simionescu CE, Stepan AE (2014) The evaluation of p16 and Ki67 immunoexpression in ameloblastomas. Rom J Morphol Embryol 55:363–367 24969987

[40] Vilgelm AE, Johnson CA, Prasad N, Yang J, Chen SC, Ayers GD, Pawlikowski JS, Raman D, Sosman JA, Kelley M, Ecsedy JA, Shyr Y, Levy SE, Richmond A (2015) Connecting the dots: therapy-induced senescence and a tumor-suppressive immune microenvironment. J Natl Cancer Inst 108:djv406. 10.1093/jnci/djv406 26719346 10.1093/jnci/djv406 PMC4849355

[41] Peng J, Lin Z, Chen W, Ruan J, Deng F, Yao L, Rao M, Xiong X, Xu S, Zhang X, Liu X, Sun X (2023) Vemurafenib induces a noncanonical senescence-associated secretory phenotype in melanoma cells which promotes vemurafenib resistance. Heliyon 9:e17714. 10.1016/j.heliyon.2023.e17714 37456058 10.1016/j.heliyon.2023.e17714 PMC10345356

[42] Karpathiou G, Hamlat M, Dridi M, Forest F, Papoudou-Bai A, Dumollard JM, Peoc’h M (2021) Autophagy and immune microenvironment in craniopharyngioma and ameloblastoma. Exp Mol Pathol 123:104712. 10.1016/j.yexmp.2021.104712 34655574 10.1016/j.yexmp.2021.104712

[43] Sathi GS, Nagatsuka H, Tamamura R, Fujii M, Gunduz M, Inoue M, Rivera RS, Nagai N (2008) Stromal cells promote bone invasion by suppressing bone formation in ameloblastoma. Histopathology 53:458–467. 10.1111/j.1365-2559.2008.03127.x 18983611 10.1111/j.1365-2559.2008.03127.x

[44] Karpathiou G, Dumollard JM, Camy F, Sramek V, Dridi M, Picot T, Mobarki M, Peoc’h M (2021) Senescence, immune microenvironment, and vascularization in cardiac myxomas. Cardiovasc Pathol 52:107335. 10.1016/j.carpath.2021.107335 33762213 10.1016/j.carpath.2021.107335

[45] Childs BG, Durik M, Baker DJ, van Deursen JM (2015) Cellular senescence in aging and age-related disease: from mechanisms to therapy. Nat Med 21:1424–1435. 10.1038/nm.4000 26646499 10.1038/nm.4000 PMC4748967

[46] Wang D, Wang H (2022) Cellular senescence in bone. Physiology. IntechOpen. 10.5772/intechopen.101803

[47] Adam A, Thomas L, Bories N, Zaharia D, Balme B, Freymond N, Dalle S (2013) Sarcoidosis associated with vemurafenib. Br J Dermatol 169:206–208. 10.1111/bjd.12268 23834124 10.1111/bjd.12268

[48] Cottrell TR, Thompson ED, Forde PM, Stein JE, Duffield AS, Anagnostou V, Rekhtman N, Anders RA, Cuda JD, Illei PB, Gabrielson E, Askin FB, Niknafs N, Smith KN, Velez MJ, Sauter JL, Isbell JM, Jones DR, Battafarano RJ, Yang SC, Danilova L, Wolchok JD, Topalian SL, Velculescu VE, Pardoll DM, Brahmer JR, Hellmann MD, Chaft JE, Cimino-Mathews A, Taube JM (2018) Pathologic features of response to neoadjuvant anti-PD-1 in resected non-small-cell lung carcinoma: a proposal for quantitative immune-related pathologic response criteria (irPRC). Ann Oncol 29:1853–1860. 10.1093/annonc/mdy218 29982279 10.1093/annonc/mdy218 PMC6096736

[49] Rawson RV, Adhikari C, Bierman C, Lo SN, Shklovskaya E, Rozeman EA, Menzies AM, van Akkooi ACJ, Shannon KF, Gonzalez M, Guminski AD, Tetzlaff MT, Stretch JR, Eriksson H, van Thienen JV, Wouters MW, Haanen JBAG, Klop WMC, Zuur CL, van Houdt WJ, Nieweg OE, Ch’ng S, Rizos H, Saw RPM, Spillane AJ, Wilmott JS, Blank CU, Long GV, van de Wiel BA, Scolyer RA (2021) Pathological response and tumour bed histopathological features correlate with survival following neoadjuvant immunotherapy in stage III melanoma. Ann Oncol 32:766–777. 10.1016/j.annonc.2021.03.006 33744385 10.1016/j.annonc.2021.03.006

[50] Haq J, Siddiqui S, McGurk M (2016) Argument for the conservative management of mandibular ameloblastomas. Br J Oral Maxillofac Surg 54:1001–1005. 10.1016/j.bjoms.2016.07.017 27599408 10.1016/j.bjoms.2016.07.017

[51] Müller H, Slootweg PJ (1985) The ameloblastoma, the controversial approach to therapy. J Maxillofac Surg 13:79–84. 10.1016/s0301-0503(85)80021-7 3858399 10.1016/s0301-0503(85)80021-7

[52] Almeida Rde A, Andrade ES, Barbalho JC, Vajgel A, Vasconcelos BC (2016) Recurrence rate following treatment for primary multicystic ameloblastoma: systematic review and meta-analysis. Int J Oral Maxillofac Surg 45:359–367. 10.1016/j.ijom.2015.12.016 26792147 10.1016/j.ijom.2015.12.016

