# Supplementary material for: Histopathologic and Molecular Insights Following the Management of Ameloblastomas via Targeted Therapies – Pathological and Clinical Perspectives
Source: Head Neck Pathol. 2024 Dec 2;18(1):129. doi: 10.1007/s12105-024-01734-2 (PMC11612134; doi:10.1007/s12105-024-01734-2)
Supplement: Supplementary file 1 — Supplementary Material 1: Supplementary Information #1 (PDF). Protocol for ameloblastoma upfront targeted therapy implemented at Sheba Medical Center, May 2023 [file 12105_2024_1734_MOESM1_ESM.pdf]

THE STATE OF ISRAEL
MINISTRY OF HEALTH
THE CHAIM SHEBA MEDICAL CENTER
Affiliated to the Tel-Aviv University
Sackler School of Medicine
TEL-HASHOMER 52621, ISRAEL

SHEBA
Tel HaShomer
City of Health

מדינת ישראל
משרד הבריאות
המרכז הרפואי המשולב ע"ש חיים שיבא
מסונף לבית הספר לרפואה ע"ש סאקלר
באוניברסיטת תל-אביב
תל-השומר 52621, ישראל

Ameloblastoma upfront targeted therapy protocol - Sheba Medical Center, May 2023

On January 2023, the Israeli Ministry of Health approved dabrafenib (Tafinlar, Novartis) with trametinib (Mekinist, Novartis) for pediatric and adult patients, with $BRAF^{v600e}$ mutated ameloblastoma.

Diagnosis

Preliminary diagnosis is performed by immunohistochemical (IHC) study - 3µ width sections from paraffin-embedded tissue are prepared and stained with: BRAF V600E antibody (clone VE1, 1:50; Spring Biosciences, Pleasanton, CA, USA), according to the manufacturer's instructions. Once confirmed by IHC, next generation sequencing (NGS) verification of the somatic mutation is performed.

Treatment protocol

Pediatric Patients - are treated upfront with BRAF inhibitor Dabrafenib (®Tafinlar, Novartis Pharmaceutical, Switzerland), daily dosing is 4.5 mg/kg/d.
Adult patients - are treated upfront by a combination of Dabrafenib and MEK Inhibitors, Trametinib (®Mekinist, Novartis Pharmaceutical, Switzerland). Daily dosing of Dabrafenib is 150 mg twice a day, daily dosing of Trametinib is 2 mg once a day.
In both groups dose reduction is performed as necessary in cases of drug intolerance. Treatment is discontinued 1 week prior to surgery.

Outcome evaluation

Base line Computerized tomography (CT) is performed, prior and in proximity to treatment initiation. Response is evaluated every 3 months by both clinical examination and magnetic resonance imaging (MRI), during treatment with targeted therapy.
Surgery is performed once achieving NADIR, or when treatment should be discontinued due to adverse toxicity (CTCAE v.5 (18). A preoperative CT is performed, for presurgical planning.

Long term follow-up

MRI scan and clinical examination every 6 months.
