# Supplementary material for: Histopathologic and Molecular Insights Following the Management of Ameloblastomas via Targeted Therapies – Pathological and Clinical Perspectives
Source: Head Neck Pathol. 2024 Dec 2;18(1):129. doi: 10.1007/s12105-024-01734-2 (PMC11612134; doi:10.1007/s12105-024-01734-2)

Mean histomorphologic scores of tumor and stroma as a factor of duration of targeted treatment per patient

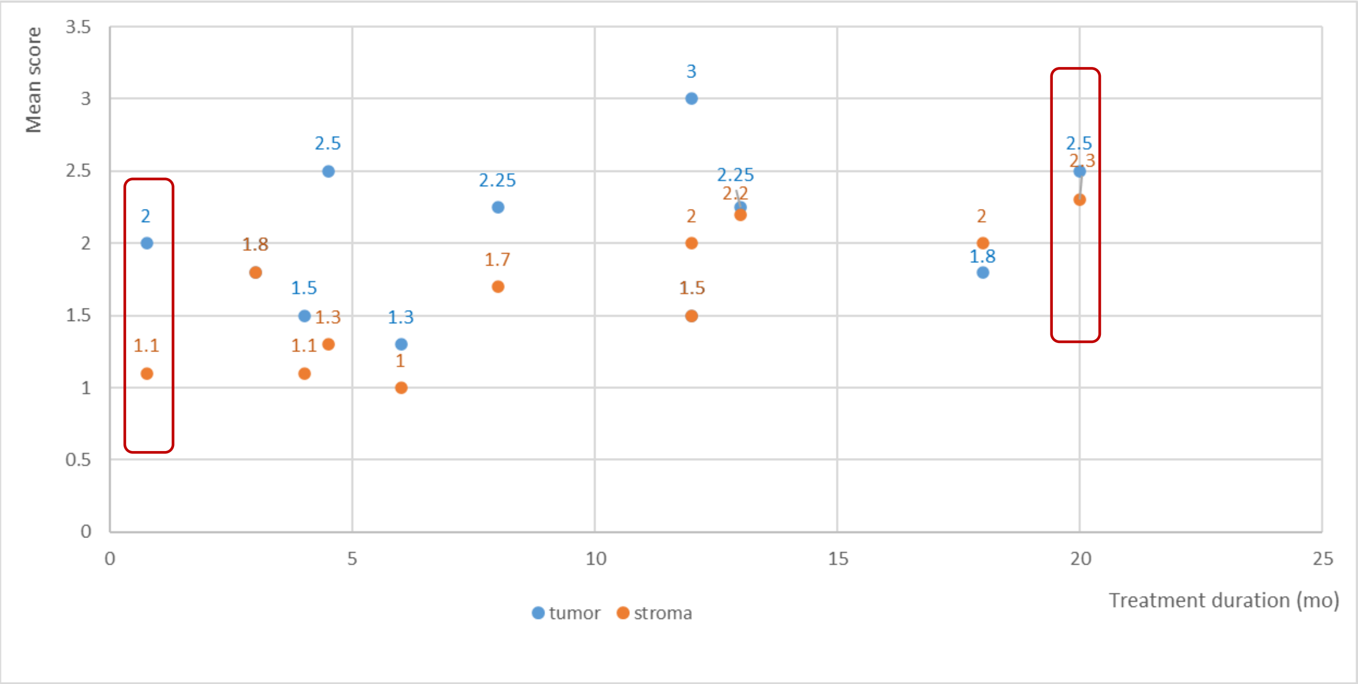

Supplement: Supplementary file 2 — Supplementary Material 2: Supplementary Information #2 (PDF). Mean histomorphologic scores of tumor and stroma as a factor of duration of targeted treatment per patient [file 12105_2024_1734_MOESM2_ESM.pdf]
